# Immune-Based Therapies for Sarcoma

**DOI:** 10.1155/2011/438940

**Published:** 2011-01-23

**Authors:** Seth M. Pollack, Elizabeth T. Loggers, Eve T. Rodler, Cassian Yee, Robin L. Jones

**Affiliations:** Fred Hutchinson Cancer Research Center, University of Washington, 825 Eastlake Avenue East, G3630, Seattle, WA 98109-1023, USA

## Abstract

Immunotherapy has shown promise in a number of tumor types, but its exact role in sarcoma remains to be defined. Advanced bone and soft tissue sarcomas are challenging diseases to treat with an unmet need for effective systemic therapy. Previous reports have suggested that immune-based treatments may be effective in sarcoma, but such approaches have not yet become part of standard clinical practice. A number of sarcoma subtypes express targets known as cancer testis antigens and hence may be excellent targets for immunotherapy. This paper will focus on the recent advances and understanding of cancer testis antigens in sarcoma and also clinical data of immunotherapeutic approaches in these diseases.

## 1. Introduction

Immunotherapy has recently had significant well-publicized successes. Placebo controlled, randomized Phase III trials have demonstrated a survival benefit for vaccine-based therapy in follicular lymphoma [1] and prostate cancer [2]. Ipilimumab, an antibody that blocks the inhibitor of T-cell activation, CTLA-4, has been shown to improve survival in patients with metastatic melanoma [3].

Progress in the systemic treatment of sarcoma has been frustratingly slow. Immunotherapy has long been discussed as a promising method for the treatment of patients with metastatic sarcoma [4, 5]. Sadly, despite a number of ambitious early phase immunotherapy trials, no immunological treatments have become part of standard clinical management. However, because of significant strides in our understanding of cancer immunology and because of progress in other disease types, immunotherapy remains a source of hope that exciting new therapies are on the horizon for patients with sarcoma. We now know that many of the most promising targets for immunotherapy are frequently expressed in certain sarcoma subtypes. Lessons learned from other diseases, such as melanoma, can guide a new generation of immunotherapy trials with the aim of preventing recurrent disease in resected sarcoma and improving the survival of patients with advanced disease.

Here we discuss some of the potential targets for immunotherapy trials with a focus on the cancer testis antigens (CTAs) and their expression in individual sarcoma subtypes. We also review prior trials of immunotherapy including nonspecific immunomodulators, vaccines, and adoptive immunotherapy.

## 2. Completed Immunotherapy Trials

Immunotherapies can be divided into the following three categories: nonspecific immunomodulation, vaccines, and adoptive cellular therapy. Nonspecific immunomodulation induces antitumor immunity without exposing the patient to a target molecule. By contrast, vaccines expose patients to antigens in order to provoke an antitumor immune response usually in the presence of adjuvant and occasionally in combination with immunomodulation [3, 6]. Some of these vaccines have been targeted to sarcoma-specific fusion products such as SYT-SSX in synovial sarcoma, whereas other vaccines are less antigen directed such as those that have used irradiated autologous tumor cells. Finally, adoptive cellular therapy involves the *ex vivo* expansion of immune effector cells (often T cells and/or NK cells) from a patient for later reinfusion. This may be nonspecific, as in the case of leukocyte-activated killer cells or tumor-infiltrating lymphocytes, or may use antigen-specific cultures *ex vivo* or genetically engineered to have tumor-directed specificity.

## 3. Nonspecific Immunomodulation

Some of the first trials demonstrating the potential of immunotherapy in cancer used high-dose interleukin-2 (IL-2) in patients with metastatic melanoma and renal cell carcinoma. Six sarcoma patients were included in these early high-dose IL-2 trials used in combination with leukocyte-activated killer cells. None of these patients responded [7]. More recently, however, high-dose IL-2 was given in a pediatric population including several patients with osteosarcoma [8]. In total, 10 pediatric patients with heavily pretreated, progressive, or metastatic solid tumors were treated with high-dose IL-2. The cohort included 4 patients with osteosarcoma and 2 patients with Ewing's sarcoma. Two of the four osteosarcoma patients had complete responses that were durable with median followup of 28 months (range 11–36 months for the 10 patients treated on study). Given that in long-term follow-up studies of adult patients with metastatic solid tumors treated with high-dose IL-2, patients who are disease-free 30 months following treatment are considered extremely unlikely to relapse [7], this pediatric study represents an encouraging finding that warrants more investigation focused on osteosarcoma. 

Muramyl tripeptide phosphatidylethanolamine (MTP) is a synthetic analogue of a bacterial cell well that has been studied clinically as a nonspecific immune modulator. Early studies demonstrated that peripheral blood mononuclear cells taken from patients following treatment with liposomal MTP demonstrated increased tumor cell killing *in vitro *compared with baseline samples [9, 10]. Furthermore, the drug was associated with increased serum levels of TNF alpha and IL-6 [10]. 

The Children's Oncology Group's Intergroup-0133 studied MTP using a 2 × 2 design. In the first randomization, patients either received or did not receive ifosfamide with a chemotherapy backbone of cisplatin, doxorubicin, and high-dose methotrexate. In the second randomization, patients either received or did not receive liposomal-MTP. Analysis of this study has been complicated; the first analysis published in 2005 showed a trend towards improved outcomes for the MTP-containing arm that was not statistically significance. With more mature followup, a 2008 report demonstrated a statistically significant improvement in overall survival with a strengthening of the event-free survival trend for the MTP-containing arm. A 2009 report in cancer suggested that improvements in outcomes may also be seen in patients with metastatic disease although this analysis was not powered to demonstrate a statistically significant benefit in either event-free or overall survival [11–13]. To date, liposomal MTP has not secured FDA approval but is available at a number of centers for compassionate use.

Alpha interferon has also been used in several sarcoma subtypes, particularly osteosarcoma, with varying success. There have been case reports of responses to interferon in osteosarcoma [14, 15] and complete responses in clear cell sarcomas [16, 17]. From 1971 to 1990, 89 consecutive patients with localized high-grade osteosarcoma received adjuvant therapy with interferon-*α*. Between 1971 and 1984, 70 patients were treated with a dose of 3 × 10^6^ IU once a day for one month, and subsequently 3 times weekly for further 17 months. Nineteen patients were treated between 1985 and 1990 with a dose of 3 × 10^6^ IU daily, with treatment extending for 2–5 years. With a median followup of 12 years (range 2–16), the observed 10-year metastases-free and sarcoma-specific survival rates were 39% and 43%, respectively. Detailed toxicity data was not available for the period following 1979, but excellent compliance with treatment implies no major additional toxicity [18, 19]. Contrasting results have been observed by other investigators. The German/Austrian cooperative study COSS-80 randomized 158 patients with localized osteosarcoma to receive methotvexate and doxorubicin with either cisplatin alone or the combination of bleomycin, cyclophosphamide, and dactinomycin. Patients were also randomized to receive or not receive 22 weeks of interferon-*β*. Interferon-*β* was commenced at week 16, consisting of 2 injections weekly for 2 weeks, then daily injections for 4 weeks and then 2 injections weekly for further 16 weeks. The dose of interferon-*β* was 100,000 U/kg. No significant difference in 30-month continuous disease-free survival was observed between patients treated with and without interferon-*β* (77% versus 73%, resp.) [20]. The differing results observed in the Scandinavian and German/Austrian studies may be due to the relatively low interferon dose and duration of therapy in the COSS-80 trial. The current European and American Osteosarcoma Study Group (EURAMOS 1) trial randomizes patients with localized osteosarcoma, who have had a good histological response to neoadjuvant chemotherapy, to receive postoperative systemic therapy consisting of methotrexate, doxorubicin, and cisplatin with or without pegylated interferon *α*-2b. The pegylated preparation of interferon *α* has an extended half life and consequently can be administered less frequently with higher dose delivery. The results of this large randomized trial will, it is hoped, define the role of interferon in the adjuvant treatment of osteosarcoma.

Ito and colleagues reported decreases in size of lung metastases in 2 out of 3 osteosarcoma patients treated with interferon. Edmonson et al. reported on a Phase II trial of recombinant interferon *α*-2a in 20 patients with advanced bone sarcomas, 17 of whom had osteosarcoma. Partial tumor regression was documented in 2 patients with osteosarcoma and one with malignant fibrous histiocytoma, for 1, 3, and 2 months, respectively. Three other patients had stable disease (each for 2 months), but all other patients had disease progression.

## 4. Targeted Immunotherapy

Potential targets for immunotherapy have been divided into five categories: mutated, shared tumor specific, differentiation antigens, overexpressed antigens, and viral antigens [21].

“Mutated” antigens involve a mutation in the cancer not present in normal tissues, thus making the target inherently specific. An example of this in sarcoma is the SYT-SSX fusion protein. This epitopes from this mutant protein have been targeted in two Phase I trials (described below). 

“Shared tumor specific” antigens are frequently expressed by a number of malignancies but rarely are expressed by normal tissue. This category includes the cancer testis antigens (described below). These antigens are highly immunogenic and are important for early development. They are frequently seen in the developing embryo but are not found in significant quantities in adults except in the testis and occasionally the placenta. 

“Differentiation antigens” are antigens involved in the normal differentiation of a specific tissue type. MART-1 is an example of this type of antigen that has been successfully targeted in melanoma. This protein is expressed as part of the normal differentiation of melanocytes and certain other cells from neural crest tissue. This differentiation antigen appears to be expressed in clear cell sarcoma as well [22]. 

“Overexpressed” targets are expressed in normal tissue but greatly overexpressed in tumors. This category includes HER2 which is frequently expressed in synovial sarcoma [23]. Some of these overexpressed antigens have been described as “universal antigens,” as they may be more uniformly expressed by tumors such as telomerase (hTERT) and survivin; these antigens may be associated with tumorigenic advantage thus targeting these antigens may circumvent the potential for outgrowth of antigen-loss variants [24, 25].

Viral antigens from viruses such as EBV have been shown to present immunogenic epitopes. This strategy may be applicable to Kaposi's sarcoma which is associated with HHV8 [26].

### 4.1. Cancer Testis Antigens

As described above, the cancer testis antigens (CTAs) are a group of proteins considered to be some of the most exciting potential targets for immunotherapy. Investigators have long sought to characterize specific tumor-associated antigens that would be considered “immunogenic,” that is, capable of inducing an immune response. Pioneering work by Thierry Boon and colleagues at the Ludwig Institute for Cancer Research in Brussels uncovered distinct antigens recognized by cytotoxic T lymphocytes (CTLs). This group first described 4 distinct antigens in mice (A, B, C, and D), two of which were products of the same gene, P1A [27, 28]. Following on the heels of this discovery, the Boon group identified the first human tumor-associated T-cell-defined antigen, MAGE-1 (Melanoma Antigen-1, subsequently renamed MAGE-A1) by screening target cells transfected with the cDNA library of a tumor line using autologous tumor reactive antigen-specific CTL. 

More T-cell-defined antigens were discovered, and MAGE-1 was eventually recognized to be part of a family of MAGE antigens which represent a broader class of antigens ultimately described by Lloyd Old as “cancer-testis” antigens. These antigens have expression restricted to germline tissues, placental trophoblasts, and a broad range of cancers. To date there are more than 70 CT gene families, many of which are being developed as T-cell targets for vaccine and adoptive cellular therapy [29].

### 4.2. Cancer Testis Antigen Expression in Specific Sarcoma Subtypes

Only a handful of articles have described cancer testis antigen expression in specific sarcoma subtypes. Complicating matters is that while all cancer testis antigens are by definition immunogenic, they are not all necessarily immunogenic for all individuals. Each CTA has epitopes described for at least one HLA type but many HLA types are quite rare. Since the class I HLA type A*02.01 is relatively common, expressed by about half of the Caucasian population, targeting A*02.01 associated epitopes in pilot immunotherapy trials for sarcoma is a reasonable approach. Some of the commonly expressed cancer testis antigens, for which A*02.01 epitopes have been identified, are NY-ESO-1, LAGE-1, PRAME, MAGE-A3, MAGE-A4, MAGE-A9, and SSX-2. The expression of these antigens in the most common sarcoma subtypes is illustrated in Table 1.

Currently, there is more data available on the expression of these antigens for synovial sarcoma than any other sarcoma subtype. It is well documented that the majority of these tumors express the cancer testis antigen NY-ESO-1, particularly those with monophasic histology where it is frequently expressed homogenously [30]. The biphasic type also expresses NY-ESO-1 in the majority of cases, although not always and occasionally these tumors may only express NY-ESO-1 in one of the biphasic compartments. Synovial sarcomas tend not to express MAGE-A1 or CT7, though little is known about the prevalence of other CT antigens in this histological subtype. One gene microarray study found that all four cases of synovial sarcoma included in that study expressed PRAME [31]. This study included 7 cases of myxoid liposarcoma and 5 nonmyxoid. All the nonmyxoid liposarcoma cases and 1 of the myxoid subtype expressed PRAME. LAGE-1 was expressed in over 70% of myxoid liposarcomas and in 60% of nonmyxoid liposarcomas [31].

One study by the Ludwig group in New York assessed CT antigen expression for a number of different sarcoma subtypes and included 6 liposarcomas [32]. Three expressed LAGE-1. 

Less is known about leiomyosarcoma and it is possible that uterine and nonuterine leiomyosarcoma have distinct patterns of CT antigen expression. In the study by Ayyoub et al., for example, four of six uterine leiomyosarcomas examined expressed MAGE-A3, while only one of the seven nonuterine leiomyosarcomas expressed MAGE-A3 [32]. Three of the six uterine leiomyosarcomas expressed NY-ESO and 2 expressed LAGE-1. No nonuterine leiomyosarcomas expressed NY-ESO and only 1 of 7 expressed LAGE-1. 

Many leiomyosarcomas, particularly uterine leiomyosarcomas, may express CTAs from the SSX family including SSX-2 which has an A*02.01 epitope. In the study of SSX antigens by Ayyoub et al. 3 of 4 expressed SSX-2 [33]. 

Among the skeletal sarcomas, osteosarcoma is known to express several CT antigens. One study of CT antigen expression in pediatric solid tumors included 9 osteosarcoma patients. All of these osteosarcoma samples expressed MAGE-A3 by real-time PCR and all but one expressed LAGE-1/NY-ESO [34]. By contrast, few of the Ewing's sarcoma patients in that study expressed cancer testis antigens.

### 4.3. Vaccine-Based Trials

A number of small trials have immunized patients against sarcoma achieving varying levels of success using a variety of different vaccines. Some of these trials have targeted well-defined antigens, others have targeted tumor lysate. In one such trial, sarcoma patients received an intradermal injection of irradiated autologous tumor cells grown in culture to vaccinate against antigens that would be released from these dying cells. Almost all of the patients also received either interferon gamma or GM-CSF as an adjuvant. An immune response was demonstrated using a delayed-type hypersensitivity (DTH) skin test against autologous tumor which converted from negative to positive in 8 of 16 evaluable patients. Median survival was 16.6 months among patients who were DTH responders compared with 8.2 months in those who were nonresponders. This was a statistically significant difference that is hypothesis generating but is of questionable causality. There were no objective responses among the study participants with measurable disease. Of note, the study included one patient with resected pulmonary metastatic disease (without measurable disease at the time of vaccination) who was disease-free over 3 years following vaccination [6]. 

One vaccine trial gave intradermal injections of dendritic cells pulsed with autologous tumor lysate [35]. Ten pediatric patients were treated; one patient with fibrosarcoma had a partial response to the treatment which included the complete regression of several sizable pulmonary sites of metastatic disease. 

The largest dendritic cell vaccine trial to date for the treatment of patients with sarcoma targeted recurrent or metastatic Ewing's sarcoma family tumors or alveolar rhabdomyosarcoma having a t(2;13) or t(11;22) translocation. Patients were treated with dendritic cells pulsed with tumor-specific peptides derived from the fusion proteins as a consolidative therapy after patients achieved a complete remission. Improved survival was seen in the group of patients receiving vaccination compared with those undergoing leukapheresis but not receiving vaccination. However, this was a nonrandomized study in which patients not receiving vaccination were more likely to have progressive disease or declining performance status [36]. In the Phase I trial of these vaccines 16 patients with bulky metastatic disease were treated, one patient had a mixed response and three patients had stabilization of disease [37].

In the posttransplant setting, a dendritic cell vaccine trial was administered to 5 children with residual tumors following autologous transplantation [38]. Three patients received dendritic cells pulsed with tumor lysate. Two patients received dendritic cells, pulsed with three synthetic tumor-specific peptides related to either the SYT-SSX2 translocation sometimes seen in synovial sarcoma or the EWS-FLI-1 fusion gene often seen in Ewing's sarcoma. One patient had a complete response that was durable for over 77 months and was ongoing at the time of the report. This was the only patient with Ewing's sarcoma receiving DCs pulsed by EWS-FLI-1-related synthetic peptides and suggests that these peptides may be worthy of further study. Two other patients had stabilization of disease but ultimately progressed. 

Several studies have used vaccines of peptide alone. One trial focused on the study drug 105AD7, a vaccineagainstthe complement regulatory protein CD55 frequently overexpressed in osteosarcoma, was able to induce cytokine production and antibody production in patients although clinical response was modest [39, 40].

A peptide encompassing the SYT-SSX fusion region of the gene resulting from the t(*X*;18) translocation has been used to vaccinate six HLA-A*24.02 positive patients. The peptide vaccine succeeded in generating peptide-specific CTLs that were successfully detected from four patients following vaccination although all patients had negative DTH skin testing. None of the patients experienced an objective clinical response although one patient's disease stabilized [41]. The same group has produced interesting *in vitro* data showing that while CTL generated to the wild-type peptide killed tumor relatively poorly (the peptide used for the vaccine), a one amino acid substituted K9I peptide (also an A*2402 associated epitope) produced CTL which killed tumor far more effectively [42]. 

There is an on-going randomized placebo controlled multicentered Phase II trial of a trivalent peptide vaccine to the gangliosides GD2, GD3, and GM2 in patients with stage IV sarcoma who have no evidence of disease following resection. These gangliosides are thought to play a role in cell adhesion and cell-cell interactions. They are usually expressed by melanomas and also may be expressed by some sarcomas [43, 44], and in Ewing's and osteosarcoma in particular [45, 46]. Moreover, soft tissue sarcoma patients frequently develop an antibody response to GD2 compared with healthy controls [47]. However, the promise of this vaccine must be tempered by the fact that a randomized trial in melanoma failed to demonstrate improvement in patient-related outcome measures [48].

### 4.4. Adoptive Immunotherapy

In adoptive immunotherapy, patients are treated with autologous lymphocytes taken from a patient and expanded *ex vivo*. Some of the most impressive clinical results have come from studies using tumor-infiltrating lymphocytes (TILs) in patients with melanoma. In these studies, tumor is taken from a patient and the lymphocytes are separated and expanded *ex vivo* and then reinfused following patient lymphodepleting conditioning. The most promising results were those patients whose condition involved an intensive regimen requiring autologous transplant with total body irradiation, cyclophosphamide and fludarabine conditioning followed by high-dose IL-2 postinfusion. The median survival for metastatic melanoma is less than a year, however, a 2-year survival rate of over 40% has been reported using this adoptive immunotherapeutic approach. It should be noted that considerable toxicity has been reported in these trials [49]. Though some early work did seem to demonstrate that TIL could often be grown in culture from patients with sarcoma, although with lower yield compared with other tumor types, little follow-up work has been done [50, 51]. However, given that a number of sarcoma subtypes do often have tumor infiltrating lymphocytes (unpublished data), this may be an area deserving further study.

Furthermore, as more has been learned about the potential targets for adoptive immunotherapy, greater interest has been given to developing T cells targeted towards specific antigens either by isolating rare tumor targeted cells from a patient's peripheral blood or by genetically modifying T cells to target a specific antigen. Given the frequent expression of CT antigens in certain sarcoma subtypes, sarcoma may be an ideal target for antigen-specific adoptive immunotherapy. The Rosenberg group at the NCI has begun treating synovial sarcoma patients with lymphocytes using a transduced T-cell receptor specific for NY-ESO-1.

## 5. Conclusion

While past attempts to use immunotherapy have failed to dramatically shift the paradigm of care for the treatment of patients with sarcoma, a great opportunity exists to shape the future. Nonspecific immunomodulation with the use of muramyl tripeptide phosphatidylethanolamine in resected osteosarcoma has shown a significant survival benefit. Other immune approaches have shown signals of potential in isolated patients with dramatic responses to immunotherapy.

A greater understanding of the immune system and the ability to harness more potent approaches to utilize the ability of the immune system to fight cancer could result in advances in the treatment of sarcoma. There remains a need for novel effective therapy in advanced soft tissue sarcoma, particularly in chemoresistant subtypes where no conventional systemic therapy is available. Emphasis on the immunological characteristics of individual sarcoma subtypes and the consequent tailoring of therapy could increase the therapeutic options available. The exact role of immunotherapy in sarcoma is yet to be delineated. It is hoped with well-designed, multiinstitutional clinical trials that this treatment approach will result in improvements in survival in this challenging group of diseases.

## Figures and Tables

**Table 1:** Selected CT antigen expression (all with A*02.01 epitopes) in selected sarcomas.

Sarcoma subtype	Reference	Method	NY-ESO-1	LAGE-1	PRAME	MAGE-A3	MAGE-A4	MAGE-A9	SSX-2
MFH/pleomorphic spindle cell	[32]	RT-PCR	1/6	0/6		1/6	0/6		
	[33]	RT-PCR							1/2
	[31]	Microarray		0/16	1/16			1/16	
Liposarcoma	[32]	RT-PCR	2/2	1/2		1/2	2/2		
	[33]	RT-PCR							1/2
Myxoid	[31]	Microarray		5/7	6/7			5/7	
Nonmyxoid	[31]	Microarray		3/5	5/5			2/5	
Leiomyosarcoma	[31]	Microarray		0/9	3/9			1/9	
Uterine leiomyosarcoma	[32]	RT-PCR	3/5	2/5		3/5	4/5		
	[33]	RT-PCR							3/4
Nonuterine leiomyosarcoma	[32]	RT-PCR	0/7	1/7		1/7	2/7		
	[32]	RT-PCR							0/1
Synovial sarcoma	[32]	RT-PCR	2/2	1/2		1/2	2/2		
	[30]	IHC	20/25						
	[31]	Microarray		3/4	4/4			3/4	
Skeletal sarcomas									
Osteosarcoma	[32]	RT-PCR	0/1	0/1		0/1	0/1		
	[33]	RT-PCR							0/1
	[34]	qRT-PCR	8/9 (NY-ESO + LAGE)			9/9	4/9		
Ewings Sarcoma	[34]	qRT-PCR	0/18 (NY-ESO + LAGE)			5/18	4/18		
Chondrosarcoma	[32]	RT-PCR	2/2	2/2		2/2	1/2		
